# Adequate Venous Drainage Does Not Exclude Cannula Malposition: Right Ventricular Perforation during Minimally Invasive Aortic Valve Replacement

**DOI:** 10.70352/scrj.cr.26-0380

**Published:** 2026-07-09

**Authors:** Kensuke Oue, Moe Kinoshita, Shintaro Okuda, Nobuo Kondo

**Affiliations:** Department of Cardiovascular Surgery, Kochi Health Sciences Center, Ike, Kochi, Kochi, Japan

**Keywords:** femoral venous cannulation, minimally invasive cardiac surgery, right ventricular perforation, cardiopulmonary bypass, transesophageal echocardiography

## Abstract

**INTRODUCTION:**

Femoral venous cannulation is widely used to establish cardiopulmonary bypass (CPB) during minimally invasive cardiac surgery (MICS). Although transesophageal echocardiography (TEE) is commonly used to guide cannula placement, cannula malposition can still occur, and preserved venous drainage may delay the recognition of cardiac injury. We report a rare case of right ventricular perforation caused by femoral venous cannulation during minimally invasive aortic valve replacement (AVR), emphasizing that adequate venous drainage does not exclude cannula malposition or cardiac perforation.

**CASE PRESENTATION:**

A 79-year-old man with severe aortic stenosis underwent minimally invasive AVR via a right mini-thoracotomy. Venous drainage was established via the right femoral vein using a 25-Fr, 55-cm HLS venous cannula with 24 side holes (BE-PVL 2555; Maquet Cardiopulmonary, Rastatt, Germany) under transesophageal echocardiographic guidance. Vacuum-assisted venous drainage was used. The guidewire was advanced toward the superior vena cava, and the cannula tip was initially considered to be positioned within the right atrium. After CPB was initiated, venous drainage became insufficient. The cannula was advanced blindly by approximately 2 cm, after which venous return improved. The valve procedure was completed uneventfully; however, pericardial bleeding became evident during volume loading before weaning from CPB. Conversion to median sternotomy revealed that the cannula had perforated the anterior free wall of the right ventricle and protruded into the pericardial cavity. The injury was repaired with a pledgeted mattress suture, and the patient recovered without further complications.

**CONCLUSIONS:**

Adequate venous drainage during CPB does not confirm correct femoral venous cannula positioning. When venous drainage is inadequate during MICS, blind advancement of the femoral venous cannula should be avoided, and cannula position should be reassessed using multiple modalities, including TEE and fluoroscopy, when available.

## Abbreviations

AVR
: aortic valve replacement
CPB
: cardiopulmonary bypass
IVC
: inferior vena cava
MICS
: minimally invasive cardiac surgery
RA
: right atrium
RV
: right ventricle
SVC
: superior vena cava
TEE
: transesophageal echocardiography
VAVD
: vacuum-assisted venous drainage

## INTRODUCTION

Peripheral femoral cannulation is frequently used to establish CPB in MICS. This approach facilitates cardiac surgery through a small thoracic incision and avoids direct central venous cannulation, but cannula-related complications can be serious.^1–4)^ Reported complications include vascular injury, venous obstruction, malposition, inadequate venous return, limb ischemia, and access-site complications.^3,4)^ RV perforation caused by a femoral venous drainage cannula is extremely rare but potentially life-threatening.^5)^

In MICS, venous cannula placement is often guided by TEE. However, TEE may not continuously visualize the entire course of the guidewire and cannula from the IVC through the RA to the SVC. This report describes RV perforation during MICS AVR after blind cannula advancement performed for poor venous drainage. The key educational message is that improved or adequate venous drainage does not exclude cannula malposition or cardiac perforation.

## CASE PRESENTATION

A 79-year-old man with symptomatic severe aortic stenosis was scheduled to undergo MICS AVR via a right mini-thoracotomy. His height was 165 cm, body weight was 53.8 kg, body surface area was 1.58 m^2^, and BMI was 19.8 kg/m^2^. After systemic heparinization, femoral arterial and venous cannulation were performed, and CPB was established before the right mini-thoracotomy. Venous drainage was obtained through the right femoral vein using a 25-Fr, 55-cm HLS venous cannula with 24 side holes over a 20-cm perforated segment (BE-PVL 2555; Maquet Cardiopulmonary, Rastatt, Germany). VAVD was used.

The guidewire was inserted through the femoral vein and directed toward the SVC under TEE guidance. The guidewire and venous cannula were confirmed to pass through the IVC–RA junction, and the cannula tip was considered to be positioned within the RA. However, continuous visualization of the entire cannula course and final tip alignment after RA entry was limited. The intraoperative TEE recordings were not available for postoperative review; therefore, retrospective image-based assessment of the precise cannula trajectory and possible RV entry could not be performed.

After the initiation of CPB, venous return was insufficient despite confirmation of circuit patency and adjustment of the venous line. To improve drainage, the venous cannula was advanced blindly by approximately 2 cm. Venous return immediately improved, and CPB flow was maintained. The AVR procedure itself was completed uneventfully through the right mini-thoracotomy. No marked hemodynamic instability was observed during CPB or before chest reopening.

**Fig. 1:**
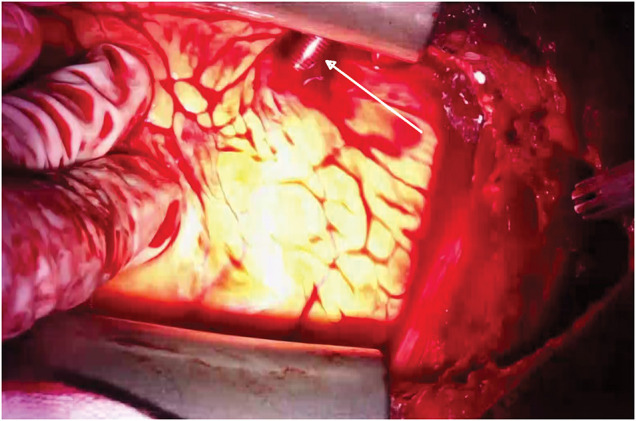
Intraoperative photograph showing perforation of the RV free wall by the femoral venous drainage cannula. The arrow indicates the cannula tip protruding into the pericardial cavity after blind advancement during CPB. CPB, cardiopulmonary bypass; RV, right ventricle

During volume loading before weaning from CPB, pericardial bleeding became apparent. Because the bleeding source could not be controlled safely through the mini-thoracotomy, the approach was converted to a median sternotomy. Intraoperative inspection revealed that the venous cannula had perforated the anterior RV free wall and was protruding into the pericardial cavity (**Fig. 1**). The cannula was carefully withdrawn under direct visualization, and the perforation site was repaired with a pledgeted mattress suture. The patient recovered without recurrent bleeding or further cannulation-related complications.

Following this event, our institutional practice was modified. Femoral venous cannulation for MICS is now performed with fluoroscopic confirmation whenever feasible, in addition to TEE, and additional advancement after CPB initiation is avoided unless the cannula course and tip position have been reassessed.

## DISCUSSION

This case provides several important lessons regarding femoral venous cannulation during MICS. First, when single femoral venous cannulation is used for MICS-AVR, the cannula tip should ideally be positioned within the SVC to ensure stable and adequate venous drainage. In the present case, although the guidewire was advanced toward the SVC under TEE guidance, the cannula tip was ultimately considered to be positioned within the RA after passing through the IVC–RA junction. In retrospect, placement within the RA alone was likely insufficient to provide stable single-cannula venous drainage and may have contributed to the initial inadequate venous return. Therefore, failure to achieve and definitively confirm SVC placement at the initial cannulation should be regarded as a contributing factor that influenced the subsequent troubleshooting process. Second, blind advancement of a large-bore femoral venous cannula without a guidewire or obturator support should be strictly avoided. In the present case, venous return improved after blind advancement of the cannula by approximately 2 cm, initially suggesting that the cannula had been repositioned successfully. However, the cannula had perforated the RV’s free wall. In retrospect, this blind advancement was the critical technical error that directly led to RV perforation. Accordingly, improvement in venous drainage should not be interpreted as confirmation of safe cannula positioning, particularly when advancement has been performed without guidewire support, obturator assistance, or direct imaging guidance.

**Fig. 2:**
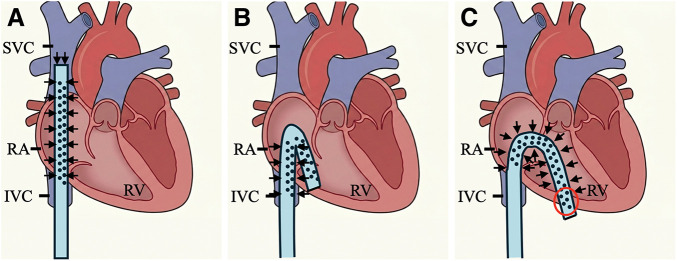
Schematic illustration of the presumed cannula trajectory, venous drainage, and mechanism of RV perforation. (**A**) Expected course of the femoral venous cannula from the IVC through the RA into the SVC, allowing effective venous drainage through the side holes. (**B**) Presumed misdirected course. The cannula entered the RA but deviated acutely toward the RV rather than advancing into the SVC. This deviation may have caused severe kinking of the cannula, compromised the effective lumen, and resulted in poor venous drainage. (**C**) Blind advancement of the cannula without guidewire or obturator support may have caused perforation of the anterior RV free wall. Subsequent release of mechanical resistance may have partially straightened the cannula and improved venous drainage under VAVD through the proximal side holes remaining within the venous lumen. Black arrows indicate venous drainage through the side holes, and the red circle indicates the presumed perforation site. IVC, inferior vena cava; RA, right atrium; RV, right ventricle; SVC, superior vena cava; VAVD, vacuum-assisted venous drainage

The paradoxical maintenance or improvement of venous drainage despite RV perforation may be explained by the configuration of the venous drainage cannula and the use of VAVD. The HLS venous cannula (Maquet Cardiopulmonary) used in this case had 24 side holes over a 20-cm perforated segment. In contrast to the expected correct trajectory along the IVC–RA–SVC axis (**Fig. 2A**), the cannula in the present case may have initially deviated acutely into the RV, undergoing severe mechanical kinking at the bend that resulted in inadequate venous return (**Fig. 2B**). We speculate that subsequent blind advancement caused the distal tip to perforate the anterior RV free wall, which paradoxically may have released the physical resistance within the ventricular chamber, partially straightened the cannula body, and relieved the kink (**Fig. 2C**). Once the kink was relieved, the effective lumen may have been restored, and venous drainage appeared to improve because the multiple proximal side holes likely remained within the venous lumen of the IVC and right-sided cardiac chambers. Under VAVD, these proximal side holes could have sustained or even enhanced pump flow despite distal perforation, creating a false sense of security and delaying recognition of the complication. The absence of obvious air entrainment may also have been related to partial sealing of the distal tip by adjacent pericardial tissue within the limited pericardial space of MICS, while venous drainage was maintained through the proximal side holes; however, this explanation remains speculative. These mechanisms may explain why adequate drainage did not exclude cannula malposition or cardiac perforation in this patient.

When venous drainage is inadequate during MICS, a systematic troubleshooting strategy should be implemented rather than resorting to blind advancement of the venous cannula. First, pump flow should be temporarily reduced if clinically feasible, and the venous drainage circuit should be checked for obstruction, clamping, air lock, kinking, or excessive negative pressure from VAVD. Second, the insertion depth, cannula orientation, and relationship between the side holes and the venous or cardiac lumen should be reassessed using TEE. Third, if the cannula position remains uncertain, further advancement should be avoided. Instead, the guidewire or obturator should be reinserted, and the cannula should be repositioned under reliable guidance, preferably with TEE and fluoroscopy when available. Fourth, if adequate drainage cannot be achieved safely through the femoral venous cannula alone, additional drainage options should be considered, including SVC cannulation, placement of a right atrial vent, or conversion to a more direct approach depending on the operative situation. Implementation of this stepwise strategy may reduce the risk of catastrophic injury associated with misdirected advancement of a large-bore venous cannula.

The limitations of TEE guidance should also be recognized. TEE is valuable for identifying the guidewire and cannula during femoral venous cannulation; however, safe placement should not rely only on confirmation that the guidewire or cannula has entered the RA from the IVC. Rather, real-time imaging should be used to verify that both the guidewire and the venous cannula traverse the RA–SVC junction and advance along the expected IVC–RA–SVC trajectory. Failure to confirm this transition may allow unrecognized deviation of the guidewire or cannula toward the RV. In the present case, the initial TEE assessment was focused on confirming passage into the RA, and subtle deviation toward the RV may have been missed. However, this could not be verified retrospectively because the intraoperative TEE recordings were not available for postoperative review. Fluoroscopy provides complementary information by allowing real-time assessment of cannula depth and alignment along the IVC–RA–SVC axis. Accordingly, fluoroscopic guidance should be strongly considered whenever additional cannula advancement becomes necessary after initiation of CPB.

Previous reports have described complications related to femoral cannulation in MICS, including vascular injury, limb ischemia, access-site complications, poor drainage, and intracardiac or caval injury^3–5)^ (**Table 1**). RV perforation remains rare, but a similar event has been reported despite the use of TEE guidance.^5)^ The present case expands the existing literature by illustrating how apparently adequate venous drainage may be maintained despite cardiac perforation and by reinforcing the practical message that preserved venous return should not reassure the surgical team when cannula position has not been definitively verified.

**Table 1:** Femoral cannulation–related complications in MICS

Complication	Presumed mechanism	Clinical consequence	References
Access-site vascular injury	Femoral arterial or venous injury during cannulation or decannulation	Bleeding, hematoma, vascular repair, limb ischemia	^3,4)^
Poor venous drainage	Malposition, kinking, insufficient cannula depth, or excessive negative pressure	Inadequate pump flow, need for repositioning	^3,4)^
Intracardiac or caval injury	Excessive or misdirected guidewire, dilator, or cannula advancement	Hemorrhage, tamponade, conversion to sternotomy	^3–5)^
RV perforation	Misdirection of the femoral venous cannula into the RV	Life-threatening pericardial bleeding	^5)^; present case
Air entrainment	Side holes outside the venous lumen or excessive negative pressure	Air in the venous line, risk of systemic air embolism	^3,4)^
Venous thrombosis	Venous stasis or endothelial injury after femoral venous cannulation	Leg swelling, pulmonary embolism risk	^4)^

MICS, minimally invasive cardiac surgery; RV, right ventricle

This case has certain limitations. The exact relationship between the cannula tip, side holes, and RV wall at the time of perforation could not be fully reconstructed. In addition, intraoperative visualization by TEE was limited, postoperative review of the intraoperative TEE recordings was not possible, and fluoroscopy was not used at the time of the event. Nevertheless, these limitations underscore the educational value of the present case and support the need for a more robust imaging strategy during femoral venous cannulation in MICS.

## CONCLUSIONS

Femoral venous cannulation during MICS can cause RV perforation if the cannula is advanced blindly to address inadequate venous drainage. Adequate or improved venous drainage during CPB does not exclude cannula malposition or cardiac perforation. Cannula position should be reassessed using TEE and, when feasible, fluoroscopy before further advancement. A structured troubleshooting strategy for inadequate venous drainage may help reduce the risk of catastrophic cannulation-related complications.

## DECLARATIONS

### Use of artificial intelligence–assisted tools

Artificial intelligence–assisted tools (ChatGPT, OpenAI) were used for language editing and improving the clarity of the manuscript. The authors critically reviewed and revised the content and take full responsibility for the final version of the manuscript.

### Availability of data and materials

All data generated or analyzed during this study are included in this published article.
